# Cationic amino acid transporter-1 (CAT-1) promotes fibroblast-like synoviocyte proliferation and cytokine secretion by taking up L-arginine in rheumatoid arthritis

**DOI:** 10.1186/s13075-022-02921-8

**Published:** 2022-10-17

**Authors:** Ying Lu, Chongbo Hao, Shanshan Yu, Zuan Ma, Xuelian Fu, Mingqing Qin, Menglei Ding, Zengguang Xu, Lieying Fan

**Affiliations:** 1 Clinical Laboratory Department, Shanghai East Hospital, School of Medicine, Tongji University, 150 Jimo Road, Pudong, Shanghai, 200120 China; 2 Shanghai East Hospital Ji’an Hospital, 80 Ji’an South Road, Ji’an City, 343000 Jiangxi Province China; 3 Research Center for Translational Medicine, Shanghai East Hospital, School of Medicine, Tongji University, 150 Jimo Road, Pudong, Shanghai, 200120 China

**Keywords:** Rheumatoid arthritis, Cationic amino acid transporter-1, L-rginine, Hyperplasia, Fibroblast-like synoviocytes

## Abstract

**Background:**

Abnormal proliferation of fibroblast-like synoviocytes (FLSs) in the synovial lining layer is the primary cause of synovial hyperplasia and joint destruction in rheumatoid arthritis (RA). Currently, the relationship between metabolic abnormalities and FLS proliferation is a new focus of investigation. However, little is known regarding the relationship between amino acid metabolism and RA.

**Methods:**

The concentrations of amino acids and cytokines in the synovial fluid of RA (*n* = 9) and osteoarthritis (OA, *n* = 9) were detected by LC–MS/MS and CBA assay, respectively. The mRNA and protein expression of cationic amino acid transporter-1 (CAT-1) were determined in FLSs isolated from RA and OA patients by real-time PCR and western blotting. MTT assay, cell cycle, apoptosis, invasion, and cytokine secretion were determined in FLSs knocked down of CAT-1 using siRNA or treated with D-arginine under normoxic and hypoxic culture conditions. A mouse collagen-induced arthritis (CIA) model was applied to test the therapeutic potential of blocking the uptake of L-arginine in vivo.

**Results:**

L-rginine was upregulated in the synovial fluid of RA patients and was positively correlated with the elevation of the cytokines IL-1β, IL-6, and IL-8. Further examination demonstrated that CAT-1 was the primary transporter for L-arginine and was overexpressed on RA FLSs compared to OA FLSs. Moreover, knockdown of CAT-1 using siRNA or inhibition of L-arginine uptake using D-arginine significantly suppressed L-arginine metabolism, cell proliferation, migration, and cytokine secretion in RA FLSs under normoxic and hypoxic culture conditions in vitro but increased cell apoptosis in a dose-dependent manner. Meanwhile, in vivo assays revealed that an L-arginine-free diet or blocking the uptake of L-arginine using D-arginine suppressed arthritis progression in CIA mice.

**Conclusion:**

CAT-1 is upregulated and promotes FLS proliferation by taking up L-arginine, thereby promoting RA progression.

**Supplementary Information:**

The online version contains supplementary material available at 10.1186/s13075-022-02921-8.

## Key message

- L-rginine was upregulated in the synovial fluid of RA patients and was transported mainly by CAT-1.
- Knockdown of CAT-1 using siRNA or inhibition of L-arginine uptake using D-arginine significantly suppressed L-arginine metabolism, cell proliferation, migration, and cytokine secretion in RA FLSs.

## Background

Rheumatoid arthritis (RA) is the most common systemic autoimmune disease characterized by chronic, symmetrical, and multiarticular inflammation, affecting nearly 0.5% to 1% of all populations in the world, and the incidence rate in China is 0.32% to 0.38%. Its high teratogenic rate is the primary cause of loss of labor force and disability in the population [1]. Although treatment for RA is constantly improving, there is still no cure for RA, only relief of symptoms. The typical pathological features of RA include abnormal hyperplasia of synovial tissue, which corrodes the surrounding tendons, ligaments, cartilage, and bone, resulting in progressive damage and deformity of joints and eventually various degrees of dysfunction [2]. Abnormal proliferation of fibroblast-like synoviocytes (FLSs) in the synovial lining layer is the primary cause of synovial hyperplasia and joint destruction [3], so inhibiting excessive proliferation of FLSs in RA represents a potential therapeutic target in RA.

Many factors in the cellular microenvironment can activate key signaling pathways of cell metabolism to promote cell growth and survival [4, 5]. Currently, the relationship between metabolic abnormalities and FLS proliferation is a new focus of investigation [6]. For example, inhibition of glycolysis alleviates FLS-mediated synovitis [7], and blocking choline kinase α alleviates synovitis by inhibiting FLS function [8]. However, little is known regarding the relationship between amino acid metabolism and RA.

L-Arginine (L-Arg) is a nonessential amino acid in healthy people. It is primarily derived from protein degradation and dietary intake and participates in energy metabolism and protein synthesis in the body. Studies in the field of oncology have confirmed that the growth of some tumor cells is significantly dependent on exogenous arginine, including kidney cancer, liver cancer, pancreatic cancer, and prostate cancer [9, 10]. In recent years, the application of arginine deiminase (ADI) to remove arginine from tumor tissues and blood to inhibit tumor growth has entered clinical research [11–13]. However, multiple clinical studies have shown that ADIs do not exert obvious therapeutic effects for the treatment of tumors. Further exploration revealed that these treatments failed because the antitumor activity of the body's own immune function was inhibited after the removal of arginine in vivo.

RA is a hyperfunctionality of the immune system, and lymphocyte activation and cytokine secretion are the primary factors that cause immune damage. RA FLSs, the main intrinsic cellular component of synovial hyperplasia of the joint, have been shown to exhibit a “tumor-like” growth characteristic: abnormal proliferation [3]. For these reasons, we investigated the molecular mechanism of RA FLS intake of arginine and attempted to identify new targeted molecules to specifically restrict the intake of arginine by FLSs to inhibit the abnormal proliferation of FLSs and reduce immune hyperactivity in RA.

L-Arginine uptake by cells occurs primarily through cationic amino acid transporters (CATs) [14], whose members include SLC7A1 (CAT-1), SLC7A2 (CAT-2), SLC7A3 (CAT-3), SLC7A4 (CAT-4), and SLC7A14. Among them, CAT-1 is expressed to varying degrees in all tissues except the adult liver and lacrimal gland [15]. However, the expression pattern of CATs in RA has not been reported thus far.

In this study, we investigated the role of L-arginine in the pathogenesis of RA and assessed the transporters through which FLSs take up L-arginine. We examined the effects of L-arginine on FLS growth in vitro and collagen-induced arthritis (CIA) mice in vivo and confirmed that CAT-1 plays an important role in L-arginine-mediated FLS proliferation and cytokine secretion, promoting the progression of RA.

## Methods

### Patient samples and ethical approval

Synovial tissue and synovial fluid were obtained from RA (*n* = 9) and osteoarthritis (OA) (*n* = 9) patients who underwent arthroscopic surgery at Shanghai East Hospital of Tongji University. All patients fulfilled the diagnostic criteria of the American College of Rheumatology (ACR) for RA and OA. None of the patients received preoperative therapy. Each patient provided their informed consent. This study was approved by the Ethics Committee of Shanghai East Hospital (No. 2020tjdx) and was conducted in accordance with the guidelines of the Declaration of Helsinki.

### Primary cell culture

Intraarticular synovial tissue obtained during arthroscopic surgery was immediately cut into 1 mm^3^ pieces under aseptic conditions. The tissue fragments were placed in a 25-cm^2^ culture flask, and a small amount of RPMI 1640 complete medium (containing 10% FBS, 1% penicillin, and streptomycin) was added. The culture flask was upside-down in a 5% CO_2_ incubator at 37 °C for 4–6 h. After the fibroblasts emerged from the tissue mass, a proper amount of complete medium was added for culture. When the primary cells grew to a fusion rate of approximately 80%, passaging was performed. The cells used in this study were cultured between 3 and 8 passages. FLSs were cultured under normoxia condition (21% O_2_, 5% CO_2_) and/or hypoxia condition (3% O_2_, 5% CO_2_) at 37 °C in BioSpherix oxygen control system. RPMI 1640 and FBS were purchased from ThermoFisher Scientific, MA, USA.

### Detection of amino acids by LC–MS/MS

Amino acid concentrations in the synovial fluid of RA and OA and L-arginine concentrations in the culture supernatant of RA FLSs were determined using LC–MS/MS. Briefly, RA FLSs transfected with CAT-1-siRNA (GenePharma, Shanghai, China) were plated at 5 × 10^6^ cells/well in 6-well plates and cultured under normoxic or hypoxic conditions for 24 h. The supernatant was collected and injected on an Agilent HILIC Plus RRHD column (Agilent, CA, USA) at 5 μl. The flow rate was held constant at 400 μl/min, and amino acids were detected.

### Cytometric bead array

A cytometric bead array (CBA)(BD Bioscience, NJ, USA) was used to detect cytokines: 2 ml of blood was intravenously collected, and the serum was separated. Fifty microliters of serum, 50 µl of a mixture of 6 cytokines, and 50 µl of PE fluorescent dye were fully mixed and reacted at room temperature for 3 h. Then, 1 mL of solution was added to each tube and centrifuged for 5 min (2000 r/min), and the supernatant was discarded. Then, another 120 µl of solution was added, shaken thoroughly, and detected 3–5 min later. A standard curve was created for each batch of kits, a dilution of the standard product was prepared according to the kit instructions, a standard tube and negative control were set during the experiment, and CBA software from American BD Company was used for analysis.

### CAT-1 knockdown in RA FLSs

To knock down CAT-1 expression in RA FLSs, we used two siRNA targeted to knockdown CAT-1 (si-CAT1-833: sense 5′-GGACACACAUGACUCUGAATT-3′, antisense 5′-UUCAGAGUCAUGUGUGUCCTT-3′; si-CAT1-1704:sense 5′-CCGUCUCUGUUUGAACAAUTT-3′, antisense 5′-AUUGUUCAAACAGAGACGGTT-3′), and a nonsilencing-siRNA (GenePharma, Shanghai, China) as a control. CAT1-siRNA or Ctrl-siRNA was transfected into RA FLSs using Lipofectamine 2000 Reagent (ThermoFisher Scientific, MA, USA) according to the manufacturer’s instructions. Forty-eight hours after transfection, RA FLSs were used to determine their function.

### Cell cycle and apoptosis assessment

RA FLSs in the logarithmic growth phase were plated at 2 × 10^5^ cells/well in 6-well plates and cultured under normoxic or hypoxic conditions for 24 h. D-Arginine (Sigma-Aldrich, MO, USA) at different concentrations (2 mM, 4 mM, 8 mM, 16 mM, and 32 mM) was added, and the control was set at the same time. After culturing for 48 h, the cells were collected and washed twice with PBS. Each sample was stained with 5 μL Annexin V and 5 μL PI solution (BD Bioscience, NJ, USA) at 4℃ for 30 min. The apoptosis rate of FLSs was detected using a BD Aria II flow cytometer and analyzed using Diva software.

Cells were collected using the same method described above, and 1 × 10^5^ FLSs were fixed in 1 mL 70% ethanol solution at 4℃ overnight. Next, the samples were centrifuged at 1000 rpm for 5 min, and the fixation solution was removed. After washing with precooled PBS 3 times, 100 μL of 100 μg/mL RNase (BD Bioscience, NJ, USA) was added to each tube. The samples were incubated with RNase at 37℃ for 30 min to degrade RNA, and then 100 μL of 50 μg/mL PI (BD Bioscience, NJ, USA) staining solution was added and incubated at 4℃ for 30 min. The cell cycle was detected using a BD FACSCalibur flow cytometer, and results were analyzed using ModFit software.

### Western blot

One hundred micrograms of synovial tissue from RA or OA patients and cultured RA FLSs transfected with siRNA or under hypoxic conditions for 48 h were added with 100 µL of RIPA cell lysate (Beyotime, Inc., Shanghai, China) to extract total cellular protein. Total protein concentration was determined using a BCA kit (Beyotime, Inc., Shanghai, China). Equal protein samples were separated on 10% polyacrylamide gels and transferred to polyvinylidene fluoride (PVDF) membranes. After the membranes were blocked in 5% skim milk for 1 h while shaking at room temperature, the primary antibody (CAT-1, 1:1000, Sigma-Aldrich, MO, USA; HIF-1α, 1:1000, *Cell Signaling Technology*, WWLP, USA) was added and incubated overnight at 4℃. We used β-actin (1:1000, Santa Cruz, TX, USA) as the loading control. The next day, the secondary antibody labeled with horseradish peroxidase was added to membranes and incubated for 1 h. The protein expression levels of CAT-1, HIF-1α, and β-actin were detected using the Odyssey Infrared Imaging System. Densitometry of the corresponding proteins was quantitatively analyzed using ImageJ software.

### MTT assay

RA FLSs in the logarithmic growth stage were plated into 96-well plates at a concentration of 5 × 10^3^ cells/mL, with 3 repeat wells in each group. Next, 200 µl cell suspension was added to each well. The 96-well plate was incubated at 37℃ for normal oxygen and 3% hypoxia culture, and an MTT test was performed on the first, second, third, and fourth days of culture as follows: 5 mg/mL MTT reagent (Promega, WI, USA) was added to each well (20 µl), and culture was continued for 4 h. The supernatant was subsequently removed, 150 µl DMSO (Sigma-Aldrich, MO, USA) was added to each well, and the plate was shaken for 15 min. Then, the OD value of each well was measured using a microplate reader (wavelength 570 nm).

### RNA extraction and real-time PCR

Total RNA of RA and OA synovial tissues was extracted using a TRIzol™ kit (ThermoFisher Scientific, MA, USA) and reverse transcribed into cDNA according to the instructions of the first-strand cDNA synthesis kit (TaKaRa, Kyoto, Japan). Real-time polymerase chain reaction (real-time PCR) was performed using SYBR Premix Ex Taq II PCR (TaKaRa, Kyoto, Japan) on ABI Q6. The reaction conditions were as follows: predenaturation at 95 °C for 30 s, denaturation at 95 °C for 5 s, and annealing at 60 °C for 30 s, for a total of 40 cycles. Dissolution curve analysis was performed, and each sample was run in triplicate. The reaction volume was 20 µL, including 2 µL cDNA, 0.8 µL primer, 10 µL SYBR Premix and 0.4 µL ROX, and ultrapure water was added to 20 µL. The 2^−ΔΔCt^ method was used for data analysis. The sequences of the primers are shown in Supplementary Table 1.

### Immunohistochemical analysis

Partial specimens of RA and OA synovial tissues were fixed in 10% formaldehyde solution (Beyotime, Inc., Shanghai, China), embedded in paraffin, sliced at a thickness of 4 µm, baked in an oven at 80 °C, dehydrated with ethanol, inactivated for endogenous peroxidase, and hydrated with citric acid buffer solution at high temperature and high pressure. Samples were then rinsed with PBS 3 times, and the primary antibody (CAT-1, Sigma-Aldrich, MO, USA) was added at 4℃ overnight. Subsequently, samples were washed with PBS 3 times, and the secondary antibody (rabbit anti-mouse IgG-HRP, Santa Cruz, TX, USA) was added. At room temperature, diaminobenzidine (DAB) (Beyotime, Inc., Shanghai, China) was added for color rendering, hematoxylin was used for contrast staining, and 1% hydrochloric acid ethanol was added for 5 min. Samples were then washed with water for 5 min before undergoing gradient ethanol dehydration (80%, 85%, 90%, 95%, 100%, 100%) and xylene transparency treatment, drying, and finally sealing with gum. The negative control was PBS instead of primary antibody.

### Cell migration assay

According to Corning® BioCoat™ Matrigel® Invasion assay kit (Corning, NY, USA), 1 × 10^5^ cells/well RA FLSs transfected with siRNA or treated with D-arginine were seeded into the upper chambers in FBS-free media, while the bottom chambers were supplied with RPMI 1640 media containing 10% FBS. After cultured under normoxic or hypoxic conditions for 24 h, non-invaded FLSs in the upper chambers were removed and invaded cells on the bottom surface were fixed with methanol, stained with crystal violet, and counted in five randomly chosen visual fields.

### Collagen-induced arthritis (CIA)

Female DBA/1 mice (*n* = 18), aged 8 weeks, were randomly divided into three groups: L-arginine-deprived feeding group, treatment group, and control group. Mice in the L-arginine-deprived feeding group were fed an arginine-free diet 2 weeks before model induction, while mice in the treatment and control groups were fed a normal diet. The treatment group was given water containing 2 mg/ml D-arginine 7 days prior to bovine type II collagen injection. CIA induction was performed based on reported methods [16]: bovine type II collagen (Sigma-Aldrich, MO, USA) was dissolved in 0.1 mol/L acetic acid to a final concentration of 2 g/L, mixed and emulsified with complete Freund’s adjuvant (V:V = 1:1) (Sigma-Aldrich, MO, USA) to prepare a type II collagen emulsion. The emulsion was intradermally injected into the tails of mice at primary and secondary immunization on days 0 and 21, respectively.

### Arthritis index (AI) evaluation

The incidence and severity of multiple arthritis lesions were recorded by AI score every 3 days beginning on day 1 of primary immunization. AI was divided into 5 levels (scored from 0 to 4): 0 = no redness or swelling; 1 = redness and swelling of the little toe joint; 2 = all joints and toes were red and swollen; 3 = redness and swelling appeared below the ankle joint; 4 = all areas were red and swollen, including the ankle joint. Arthritis induction was considered successful if at least one foot was red and swollen, including the ankle joint.

### Statistical analysis

All data mentioned above are presented as the mean ± SD of at least 3 independent experiments. The results were analyzed using the statistical software SPSS 17.0 and GraphPad Prism 8 software. The quantitative data of two groups were analyzed using two-tailed Student’s *t*-tests, and comparisons among three or more groups were conducted using one-way ANOVA. A correlation analysis was performed between amino acids and cytokines using Pearson’s correlation method. A *p*-value less than 0.05 was considered statistically significant.

## Results

### L-Arginine is upregulated in the synovial fluid of RA and is positively correlated with the elevation of cytokines

**Fig. 1:**
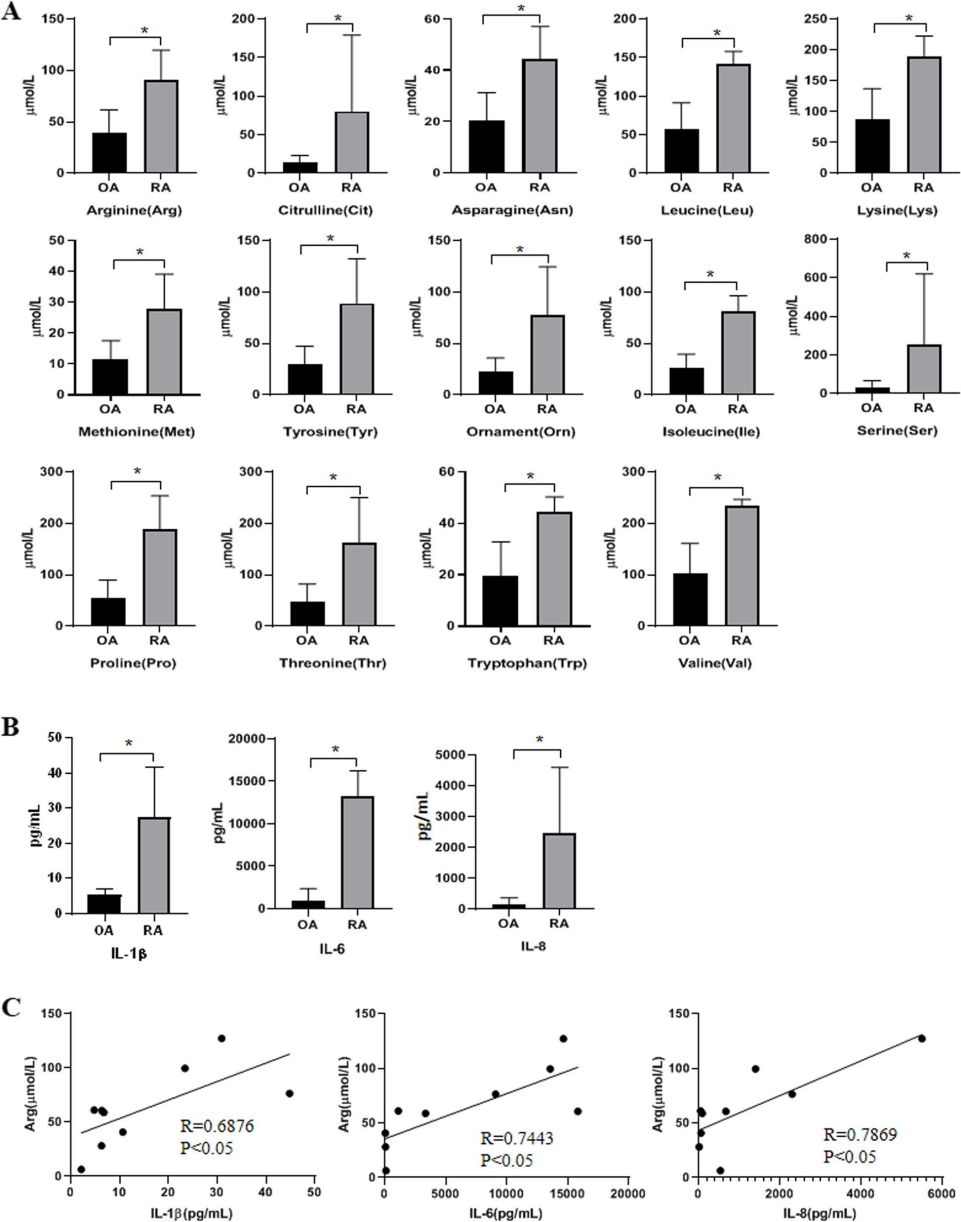
L-Arginine is upregulated in the synovial fluid of RA and is positively correlated with the elevation of cytokines. **a** The concentrations of amino acids in the synovial fluid of RA and OA were detected by LC–MS/MS. **b** The concentrations of cytokines were detected in the synovial fluid of RA and OA by CBA assay. **c** Pearson’s correlation analysis of the relationship between cytokines and L-arginine levels in the synovial fluid of RA patients. Data are shown as the mean ± SD from three independent experiments and each dot plot represents an individual sample. *P < 0.05

LC–MS/MS was performed to determine the concentrations of 18 amino acids in synovial fluid of RA and OA (*n* = 9). The results revealed that the concentrations of 14 amino acids (L-arginine, L-citrulline, L-asparagine, L-leucine, L-lysine, L-methionine, L-tyrosine, L-ornament, L-isoleucine, L-serine, L-proline, L-threonine, L-tryptophan and L-valine) in the synovial fluid of RA were significantly higher than those in OA (Fig. 1a). The concentrations of the other 4 amino acids (L-alanine, L-glutamic acid, glycine and L-histidine) showed no significant differences between RA and OA synovial fluid (Supplementary Fig. 1a). Next, we assessed the concentrations of cytokines (IL-1β, IL-2, IL-4, IL-5, IL-6, IL-8, IL-10, INF-γ, INF-α, TNF-α, IL-12P70) and found that IL-1β, IL-6 and IL-8 were elevated significantly in the synovial fluid of RA compared to OA (Fig. 1b). However, there were no significant differences in other cytokines (IL-2, IL-4, IL-5, IL-10, INF-γ, INF-α, TNF-α, IL-12P70) between RA and OA (Supplementary Fig. 1b). Furthermore, we analyzed the relationship between amino acids and cytokines in the synovial fluid of RA patients and found that only L-arginine was positively correlated with elevated IL-1β, IL-6 and IL-8 levels (Fig. 1c).

### The arginine transporter CAT-1 is overexpressed in RA FLSs and is elevated under hypoxia

**Fig. 2:**
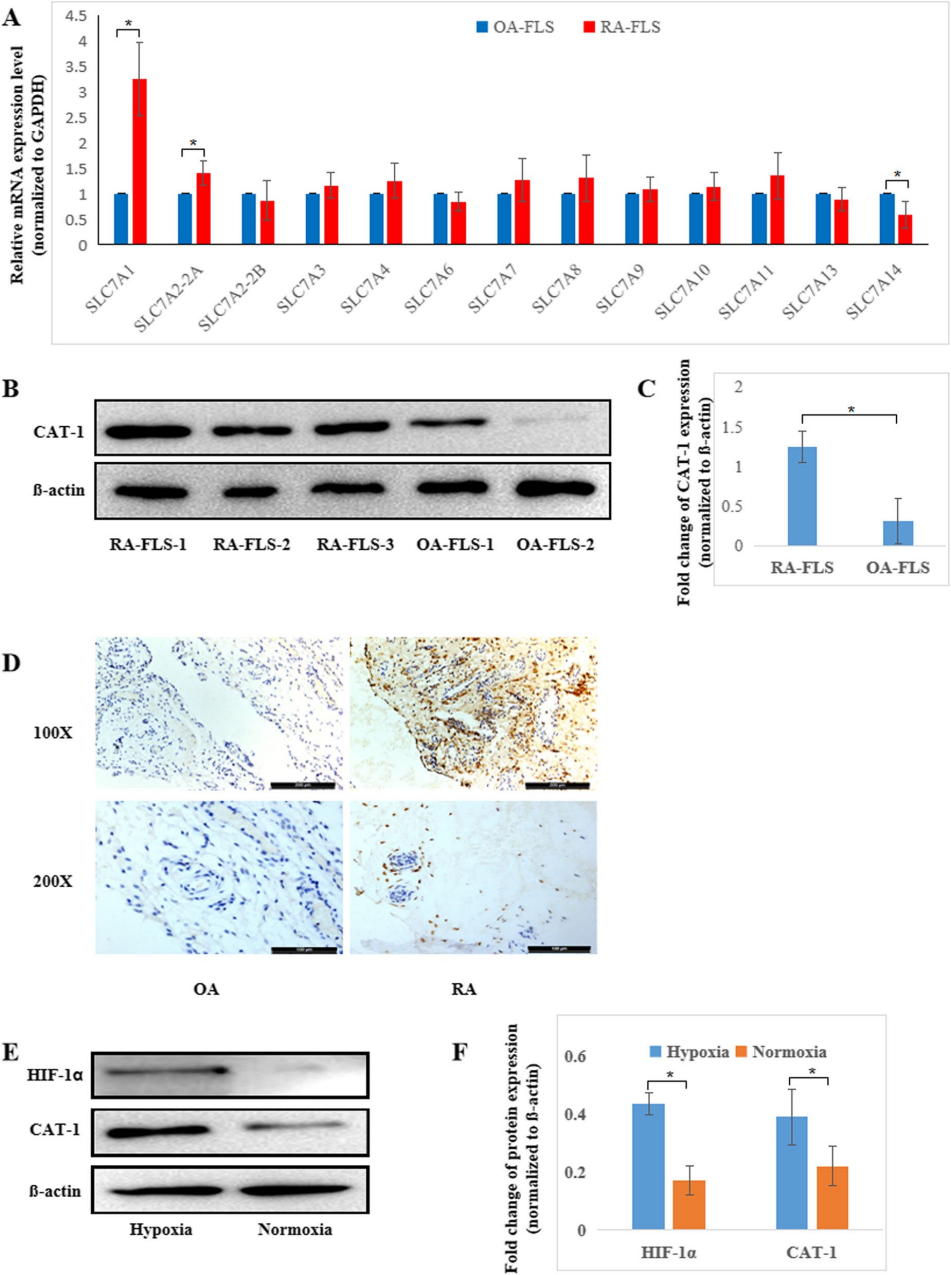
The arginine transporter CAT-1 is overexpressed in RA FLSs and is elevated under hypoxia. **a** mRNA expression of SLC7 family transporters in 9 RA and OA synovial tissue samples were detected by RT-PCR, and GAPDH was used as the internal control. The *P*-value was calculated by Student’s test. **b** Representative image of CAT-1 protein expression in RA FLSs and OA FLSs by Western blots. **c** The relative protein levels of CAT-1 in RA FLSs and OA FLSs were analyzed, and β-actin was used as the internal control. **d** Representative images of the protein expression of CAT-1 in RA and OA synovial tissue. **e** The protein expression of HIF-1α and CAT-1 in RA FLSs cultured under normoxic or hypoxic conditions for 24 h were analyzed by Western blot. **f** The relative protein levels of HIF-1α and CAT-1, and β-actin was used as the internal control. The data are expressed as the mean ± SD of three independent experiments. *P < 0.05

Because abnormal proliferation of FLSs is the primary cause of synovial hyperplasia and joint destruction in RA, we used real-time PCR to analyze the mRNA expression of common arginine transporters (SLC7 family) in RA and OA synovial tissue samples (*n* = 9). Among the multiple amino acid transporters that exist in RA FLSs, SLC7A1 (also called cationic amino acid transporter-1, CAT-1) and SLC7A2-2A have been shown to be upregulated compared to OA. Nevertheless, SLC7A14 was downregulated. There were no significant differences in other amino acid transporters (Fig. 2a). Next, we examined the protein levels of CAT-1 and SLC7A2-2A in FLSs isolated and cultured from RA and OA synovial tissue by western blot. The results showed that CAT-1 expression was significantly higher in RA FLSs than in OA FLSs (Fig. 2b, c). SLC7A2-2A could not be detected (data not shown). Consistent with the western blot results, the protein expression of CAT-1 in RA synovial tissue was higher than that in OA synovial tissue, as shown in Fig. 2d.

Furthermore, as reduced oxygen levels in the synovium of RA have been demonstrated and 3% oxygen has been confirmed to represent the joint environment in RA, we cultured RA FLSs under 3% O_2_ for 24 h and determined the protein expression of CAT-1. The results showed that CAT-1 was upregulated under hypoxia, along with the expression of hypoxia-inducible factor-α (HIF-α) (Fig. 2e, f).

### CAT-1 promotes FLS cell growth and inhibits apoptosis by transporting L-arginine in RA

**Fig. 3:**
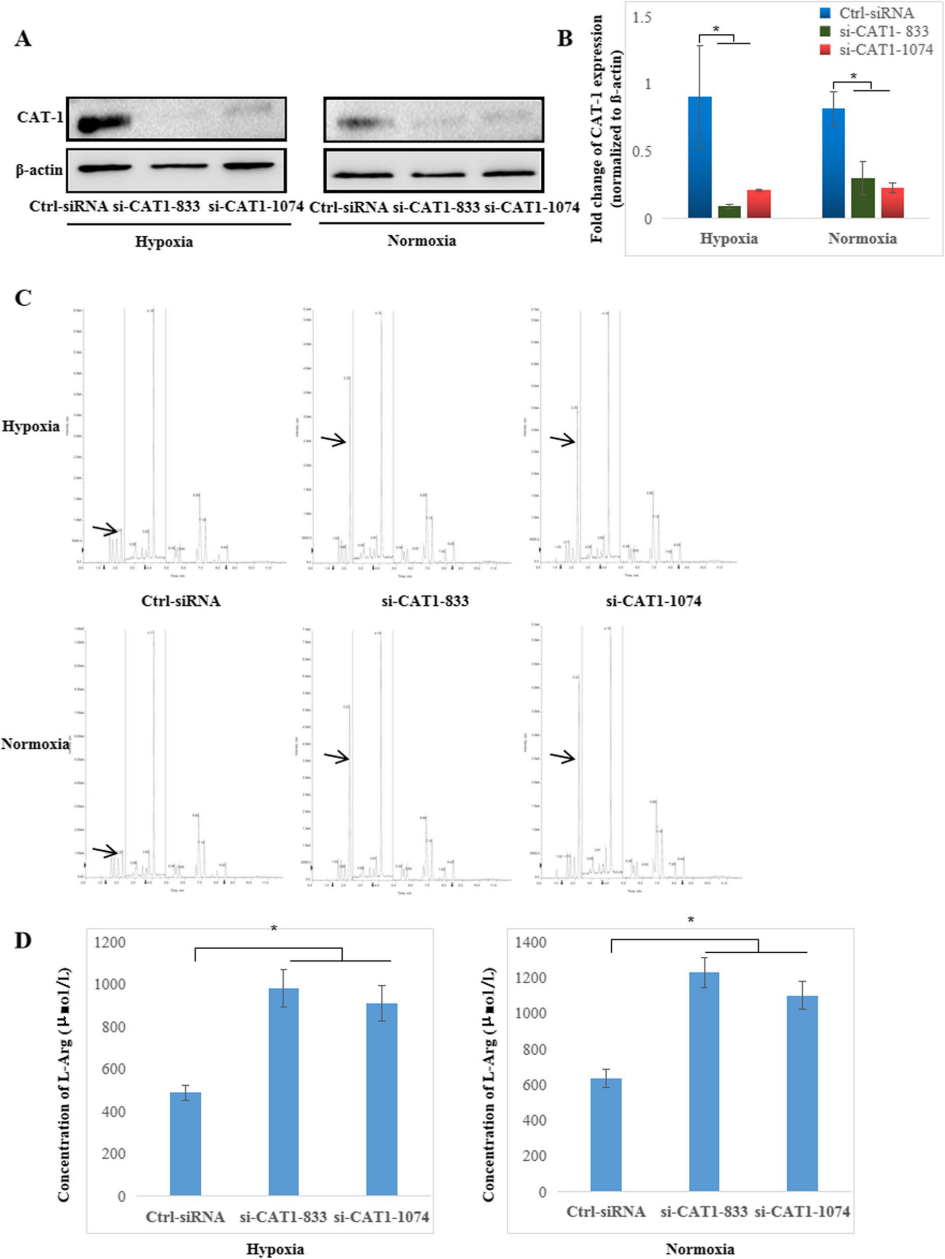
Knockdown CAT-1 inhibits L-arginine uptake in RA FLSs. **a** Decreased protein expression of CAT-1 in RA FLSs transfected with CAT1-siRNA and cultured under normoxic or hypoxic conditions were detected by western blots. **b** The relative protein levels of CAT-1 in RA FLSs transfected with CAT1-siRNA were analyzed, and β-actin was used as the internal control. **c** Representative peak figures of L-arginine in the supernatant of RA FLSs transfected with CAT1-siRNA and cultured under normoxic or hypoxic conditions were detected by LC–MS/MS. **d** The concentration of L-arginine in the supernatant of RA FLSs transfected with CAT1-siRNA and cultured under normoxic or hypoxic conditions. The data are expressed as the mean ± SD of three independent experiments. *P < 0.05

To determine the biological function of CAT-1 in RA FLSs, we performed cell proliferation assays, apoptosis assays and cell cycle analysis of FLSs treated with CAT-1-siRNA or Ctrl-siRNA and cultured under normoxic or hypoxic conditions. Western blot analysis revealed that the protein expression of CAT-1 in RA FLSs after knockdown of CAT-1 with siRNA was significantly decreased under both normoxic and hypoxic conditions (Fig. 3a, b). LC–MS/MS was used to determine L-arginine concentration in the culture supernatant of RA FLSs and revealed that its concentrations were increased in the culture supernatant of RA FLSs by silencing CAT-1 with siRNA under both normoxic and hypoxic conditions, indicating that CAT-1 is the primary transporter for L-arginine in RA FLSs (Fig. 3c, d).

**Fig. 4:**
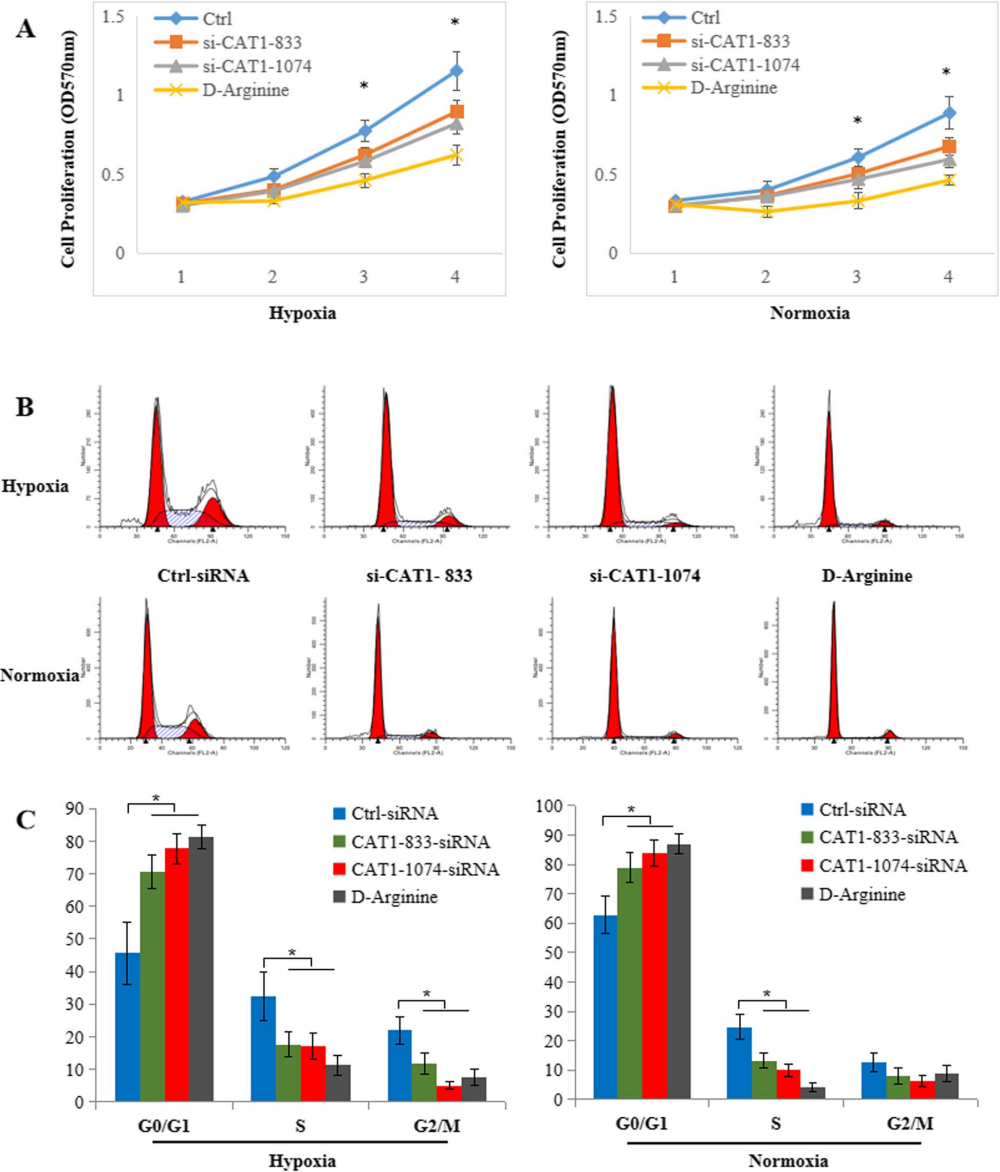
Knockdown CAT-1 suppresses cell proliferation. **a** The growth curve of RA FLSs transfected with CAT1-siRNA or treated with D-arginine and cultured under normoxic or hypoxic conditions, as determined by MTT assay. **b** Cell cycle analysis G_1_/S transition of RA FLSs transfected with CAT1-siRNA or treated with D-arginine and cultured under normoxic or hypoxic conditions, as determined by flow cytometry. **c** Quantification of cell cycle analysis. The data are expressed as the mean ± SD of three independent experiments. *P < 0.05

D-arginine is a competitive inhibitor of L-arginine. MTT assays were used to assess cell growth after CAT-1 knockdown or treatment with D-arginine. As shown in Fig. 4a, RA FLSs transfected with CAT-1-siRNA or treated with D-arginine displayed significant growth inhibition compared to those transfected with Ctrl-siRNA. We also measured cell cycle progression using flow cytometry analysis and found that RA FLSs transfected with CAT-1-siRNA or treated with D-arginine exhibited a significant increase in G0/G1 phase and a significant decrease in the S phase compared to those transfected with Ctrl-siRNA (Fig. 4b, c).

**Fig. 5:**
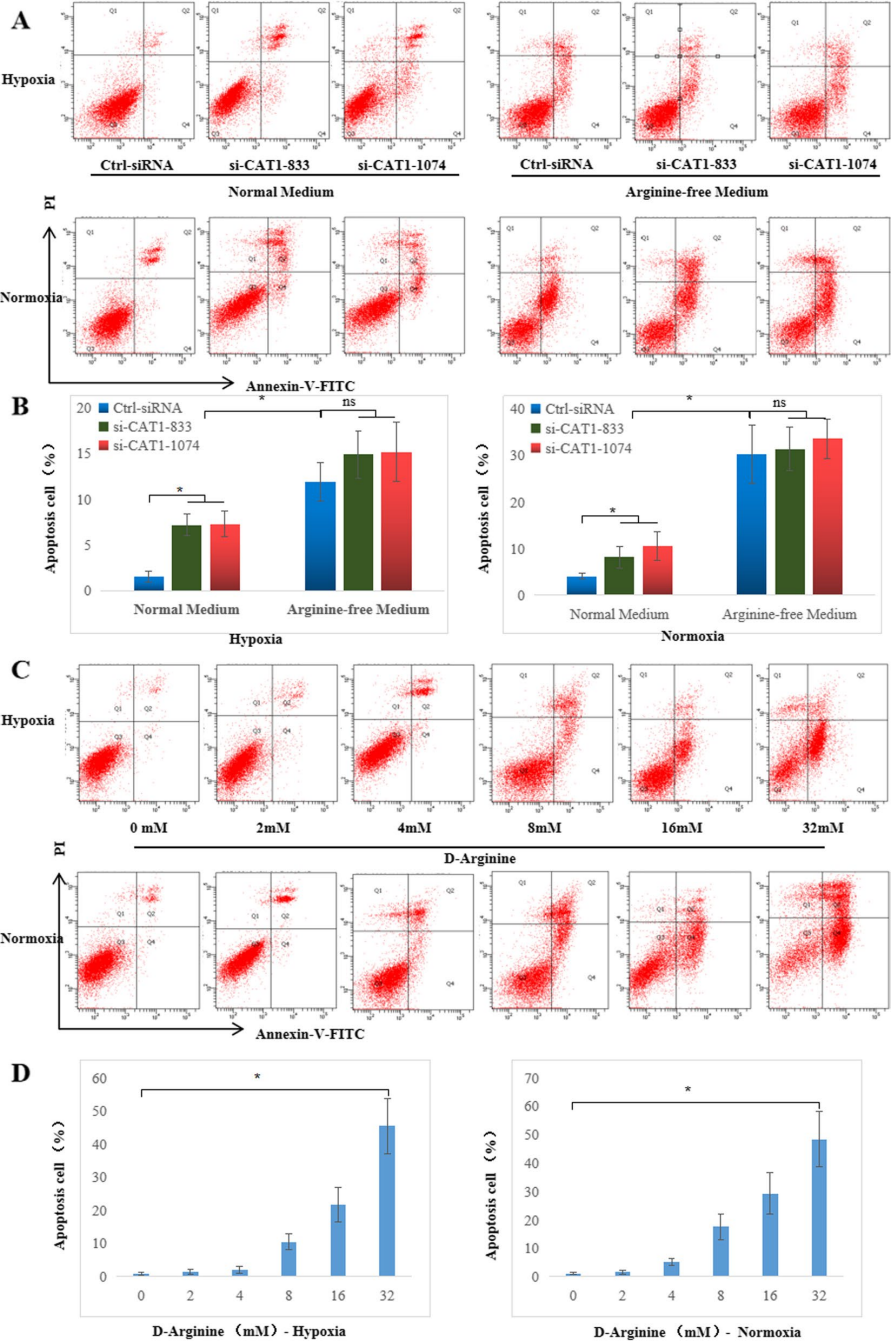
Knockdown CAT-1 or inhibition of L-arginine uptake increased cell apoptosis in a dose-dependent manner. **a** The cell apoptosis of RA FLSs transfected with CAT1-siRNA cultured in normal RPMI 1640 medium or L-arginine-free medium was stained with Annexin-V/PI and examined by flow cytometry. **b** The quantification of apoptotic cells identified as PI-negative and Annexin-V-positive staining. **c** The cell apoptosis of RA FLSs treated with increasing D-arginine concentrations was stained with Annexin-V/PI and examined by flow cytometry. **d** The quantification of apoptotic cells. The data are expressed as the mean ± SD of three independent experiments. *P < 0.05

Whether CAT-1 affects cell apoptosis was also determined in normal and arginine-free medium by flow cytometry analysis. Interestingly, the percentage of early apoptotic cells significantly increased in the CAT-1-siRNA-treated RA FLSs compared to the Ctrl-siRNA-treated cells in normal medium but not in arginine-free medium in the normoxic or hypoxic environment. Moreover, the percentage of early apoptotic cells significantly increased in arginine-free medium compared with normal medium (Fig. 5a, b). Furthermore, we investigated the effect of different concentrations of D-arginine on apoptosis, and as expected, apoptotic cells increased significantly with increasing D-arginine concentrations (Fig. 5c, d). These findings indicated that CAT-1 is required for cell proliferation, which occurs through transporting L-arginine in RA FLSs.

### CAT-1 enhances the migration of RA FLSs and promotes cytokine secretion

**Fig. 6:**
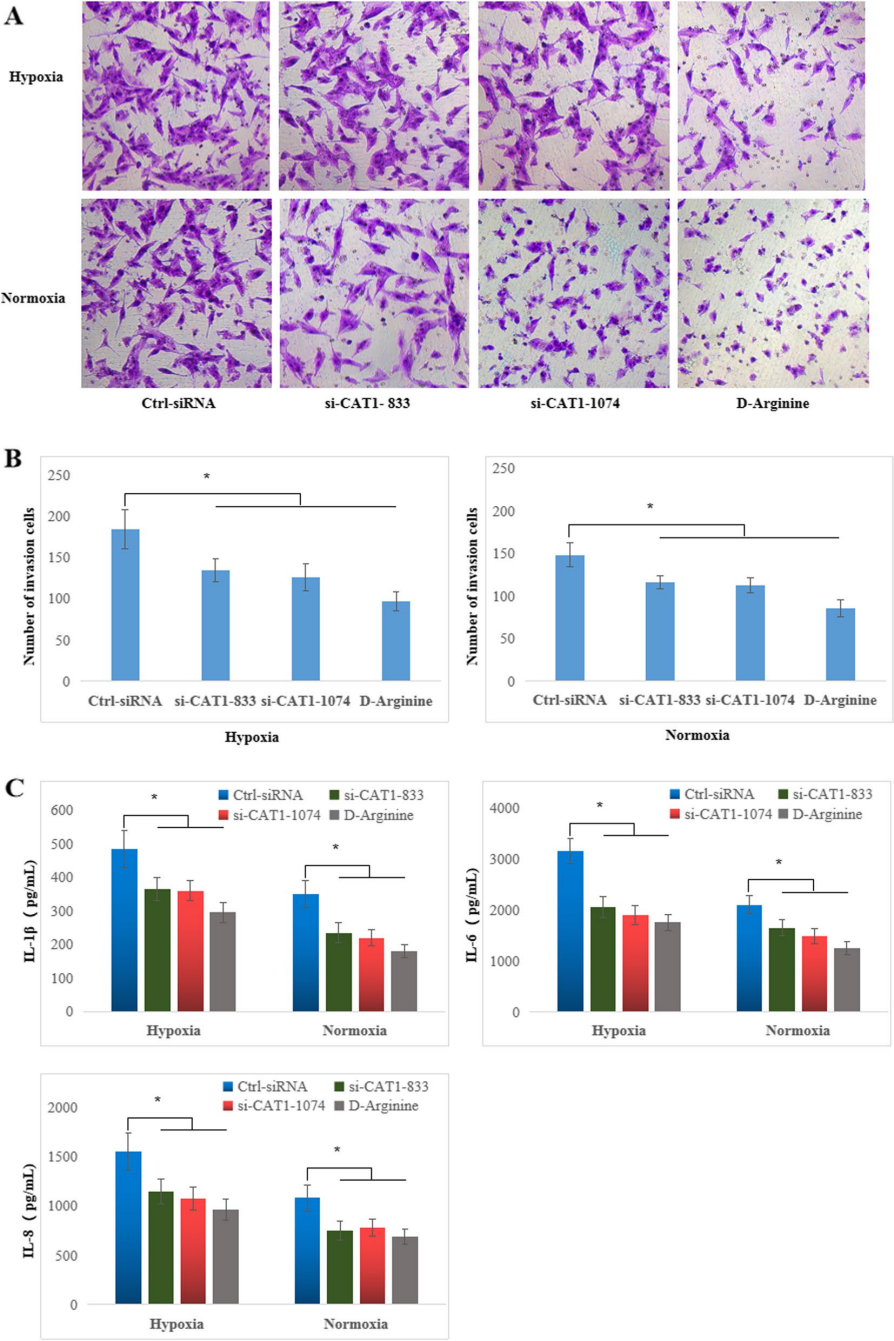
Knockdown CAT-1 reduced migration and cytokine secretion of RA FLSs. **a** The migratory abilities of RA FLSs transfected with CAT1-siRNA or treated with D-arginine and cultured under normoxic or hypoxic conditions were evaluated by transwell chamber assays. Representative fields of migrated cells are shown. **b** Quantification of cells. **c** Inflammatory cytokines released in cultural supernatant of RA FLSs transfected with CAT1-siRNA or treated with D-arginine and cultured under normoxic or hypoxic conditions were detected by CBA assay. The data are expressed as the mean ± SD of three independent experiments. *P < 0.05

To determine whether CAT-1 plays a role in RA FLS migration, we performed Transwell assays and demonstrated that CAT-1 knockdown or treatment with D-arginine significantly reduced cell migration under both normoxic and hypoxic culture conditions compared with Ctrl-siRNA treatment (Fig. 6a, b). Furthermore, IL-1β, IL-6, and IL-8 were assessed to evaluate the effect of CAT-1 on RA FLSs cytokine secretion. As shown in Fig. 6c, knockdown of CAT-1 or treatment with D-arginine reduced the concentrations of IL-1β, IL-6, and IL-8 in the supernatant of RA FLSs compared to Ctrl-siRNA-treated cells. These results indicated that CAT-1 plays an important role in RA FLS migration and cytokine secretion in vitro.

### Inhibition of L-arginine uptake suppresses CIA mouse induction

**Fig. 7:**
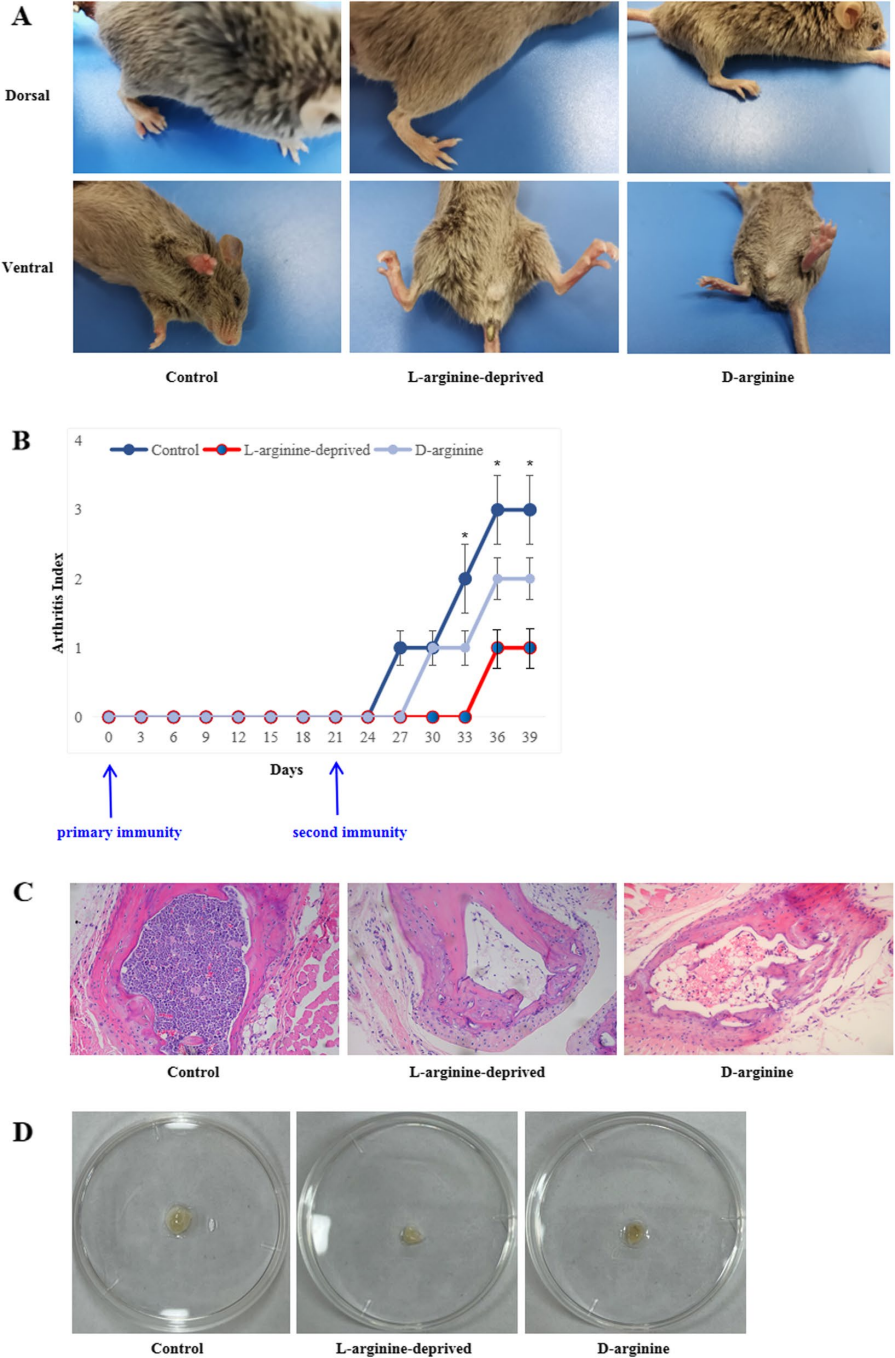
Inhibition of L-arginine uptake suppresses CIA mouse induction. **a** Swellings of paws and joints were more apparent in control mice (*n* = 6) compared with L-arginine deprivation (*n* = 6) and D-arginine addition (*n* = 6) mice. **b** Average arthritic index of control, L-arginine deprivation, and D-arginine addition mice were evaluated. **c** H&E staining analysis of inflammatory cell infiltration in the joint from each group. **d** Inguinal lymph nodes from each group were collected and photographed. The data are expressed as the mean ± SD of three independent experiments. *P < 0.05

We next assessed the role of L-arginine in a collagen-induced arthritis mouse model. Bovine type II collagen was injected into the caudal vein of DBA/1 mice to induce arthritis, and the effect of L-arginine deprivation and D-arginine addition on CIA was evaluated. As shown in Fig. 7a and b, we found that L-arginine deprivation and D-arginine addition dramatically inhibited the induction of arthritis in these mice compared to the control group. The arthritis index in the CIA control group was higher than that in the L-arginine deprivation and D-arginine treatment groups. Further histopathological data confirmed that the control group with CIA symptoms was accompanied with a large number of inflammatory cell infiltration in the joint and inguinal lymph node enlargement (Fig. 7c, d).

## Discussion

Studies have found that L-arginine plays a very important regulatory role in the function of immune cells in humans [17–19]. Recently, it was reported that increasing the concentration of L-arginine alters the metabolic state of T cells from glycolysis to oxidative phosphorylation, improving the survival rate of T cells and facilitating the production of an antitumor immune response, and immune function is weakened by the deprivation of L-arginine [20]. L-arginine has also been used to improve the immunity of cancer patients to treat tumors [21]. L-arginine removal in the treatment of cancer is a double-edged sword. While inhibiting the growth of tumor cells, it also greatly inhibits the ability of autoimmune cells to remove tumor cells. Therefore, the strategy of arginine removal in the treatment of cancer remains to be validated. RA is an autoimmune disease caused by immune hyperfunctionality. In the pathogenic joint cavity, there is not only an abnormal proliferation of FLSs with “tumor-like” characteristics that erode the joint but also inflammatory responses caused by infiltration of a large amount of inflammatory information and secreted inflammatory factors. Therefore, limiting L-arginine absorption in RA patients may be more promising than as a treatment for tumors because blocking L-arginine absorption can not only inhibit the proliferation of FLSs but also relieves immune hyperfunction.

Surprisingly, our results confirmed higher concentrations of all 14 amino acids in RA than in OA, but only elevated concentrations of L-arginine were positively associated with elevated levels of the inflammatory cytokines IL-1β, IL-6, and IL-8. This may be because the abnormal proliferation of FLSs in RA requires a large amount of nutrients, and the high concentration of L-arginine promotes the proliferation of FLSs and the activation of immune cells: the amount of cytokines secreted by the increased FLSs increases, and the activation of immune cells also secretes many cytokines. Furthermore, previous studies have shown that in addition to IL-1β, IL-6, and IL-8, other cytokines, such as TNF-α and IFN-γ, are also elevated in RA articular fluid [22–25], but in this study, there was no significant difference compared to the concentrations observed in OA synovial fluid. This may be due to the small sample size (only 9 samples were used in this study), or perhaps different control samples should have been selected. However, IL-1β, IL-6, and IL-8 are the most common cytokines in the inflammatory response of RA, so they are used to represent the inflammatory state of RA.

Amino acids cannot pass freely through the cell membrane and must be transported from the extracellular space to the intracellular space by specific transporters. Arginine transporters are primarily cationic amino acid transporters, including SLC7A1 (CAT-1), SLC7A2 (CAT-2), SLC7A3 (CAT-3), SLC7A4 (CAT-4), and SLC7A14. Other proteins, SLC7A5–SLC7A13, are the main L-type amino acid transporters [15]. To determine which transporter RA FLSs primarily transport arginine, we detected the SLC7 family transporter and found that the mRNA expression of SLC7A1 (CAT-1) and SLC7A2-2A in RA FLSs was increased, but the increase in CAT-1 was significant. Further WB and immunohistochemistry results confirmed that the protein expression of CAT-1 in RA FLSs was significantly increased. Therefore, we believe that CAT-1 is the primary arginine transporter of RA FLSs.

Studies have confirmed that oxygen levels in the synovial cavity of patients with inflammatory arthritis are lower than that in the control population, and the low oxygen level in the synovial cavity of inflammatory joints is related to microvascular proliferation, synovial hyperplasia and oxidative damage [26–28]. In this study, 3% O2 was used as a hypoxic culture condition to simulate the microenvironment in the RA joint cavity, which is consistent with the results of other studies [29]. Hypoxia promoted the proliferation of RA FLSs and inhibited apoptosis. Some researchers believe that the reason for RA’s hypoxic environment is that the abnormal proliferation of cells in the joint cavity consumes a large amount of oxygen, resulting in local hypoxia [30]. Hypoxia promotes angiogenesis, inhibits apoptosis, and further promotes cell proliferation, forming a positive feedback loop that aggravates the hypoxic microenvironment. To our surprise, inhibition of L-arginine uptake by RA FLSs in both normoxic and hypoxic environments significantly reduced FLS proliferation, invasion, and cytokine secretion. These results indicate that the function of L-arginine in RA FLSs is independent of the hypoxic environment.

Normal human cells utilize L-arginine for metabolism through the nitric oxide synthase and ornithine-polyamine pathways [31]. Arginine is metabolized by arginase (ARG) to produce ornithine, which is metabolized by ODC to produce polyamine. Putrescine plays an important role in tumorigenesis and cell proliferation [32]. Arginine is also a substrate of nitric oxide synthase (NOS), whose metabolic products are nitric oxide (NO) and citrulline. Studies have found that hypoxia can cause changes in cell metabolism and induce the generation of iNOS. Under the catalysis of iNOS, the generated NO can stabilize the activation state of hypoxia-inducible factor HIF, promoting cell proliferation [33]. HIF, a nuclear transcription factor that regulates the expression of cellular oxygen balance and hypoxic response genes, is increased in hypoxic conditions and has been demonstrated to inhibit apoptosis and promote cell proliferation and survival through multiple pathways, such as p53 [34–36]. Moreover, it was reported that in mouse RA models, NO content is significantly increased and promotes synovial hyperplasia and inflammatory damage in rheumatoid arthritis [37]. However, the metabolic pathway through which L-arginine promotes cell growth in RA FLSs has not been reported thus far, which is what we plan to investigate next.

CAT-1 is a membrane protein, so it may be an ideal target for monoclonal antibodies for targeted therapy. Although normal tissues express basal levels of CAT-1, RA FLSs significantly overexpress CAT-1, indicating that the anti-CAT-1 monoclonal antibody would be sensitive in RA FLSs but have little effect on normal cells. Monoclonal antibodies targeting CAT-1 not only inhibit L-arginine uptake by RA FLSs and inhibit synovial hyperplasia but also may suppress disease progression by weakening T cell immunity.

## Conclusions

Our experiments indicated that CAT-1 is upregulated and promotes FLS proliferation by taking up L-arginine, thereby promoting RA progression. Inhibition of L-arginine uptake reduced RA FLS growth and cytokine secretion in vitro and suppressed arthritis induction in vivo. These findings highlight the clinical and therapeutic utility of CAT-1 to target the constitutive anabolism of RA FLSs for treatment.

## Supplementary Information

**Additional file 1.**

**Additional file 2: Table S1.** Primers for qRT-PCR.

## Data Availability

The datasets used and analyzed during the current study are available from the corresponding author on reasonable request.

## Abbreviations

ACR: American College of Rheumatology
ADI: Arginine deiminase
AI: Arthritis index
CAT-1: Cationic amino acid transporter-1
CBA: Cytometric bead array
CIA: Collagen-induced arthritis
FLS: Fibroblast-like synoviocytes
OA: Osteoarthritis
PVDF: Polyvinylidene fluoride
RA: Rheumatoid arthritis
