# Supplementary figures and images for: Cationic amino acid transporter-1 (CAT-1) promotes fibroblast-like synoviocyte proliferation and cytokine secretion by taking up L-arginine in rheumatoid arthritis
Source: Arthritis Res Ther. 2022 Oct 17;24:234. doi: 10.1186/s13075-022-02921-8 (PMC9575222; doi:10.1186/s13075-022-02921-8)

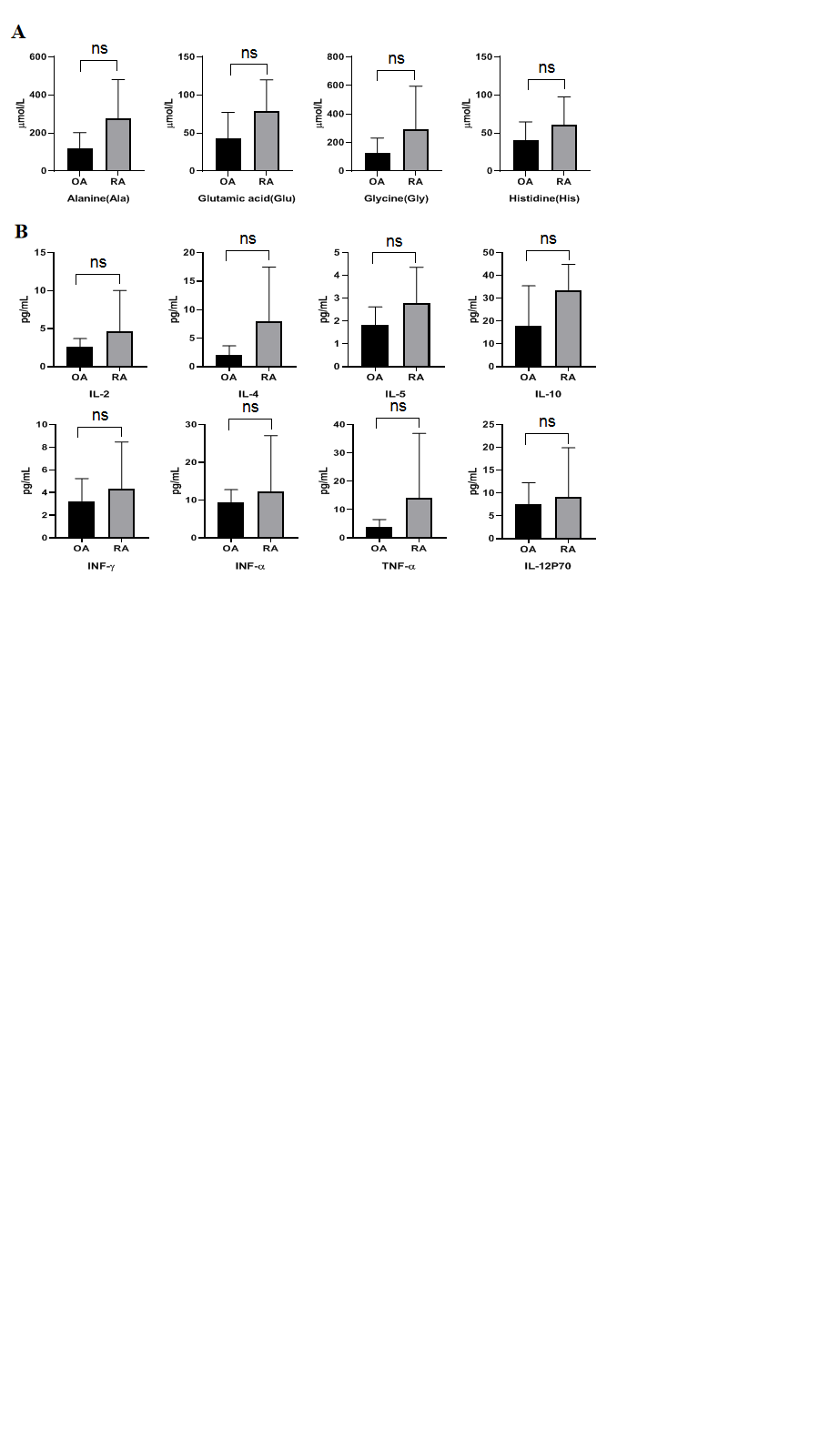

Supplement: Supplementary file 1 — Additional file 1. [file 13075_2022_2921_MOESM1_ESM.tif]
