# Supplementary material for: Cationic amino acid transporter-1 (CAT-1) promotes fibroblast-like synoviocyte proliferation and cytokine secretion by taking up L-arginine in rheumatoid arthritis
Source: Arthritis Res Ther. 2022 Oct 17;24:234. doi: 10.1186/s13075-022-02921-8 (PMC9575222; doi:10.1186/s13075-022-02921-8)
Supplement: Supplementary file 2 — Additional file 2: Table S1. Primers for qRT-PCR. [file 13075_2022_2921_MOESM2_ESM.docx]

**Supplementary Table S1**. Primers for qRT-PCR

| Gene |  | 5’- 3’ Sequence |
| --- | --- | --- |
| GAPDH | forward | GAGTCAACGGATTTGGTCGTATTG |
|  | reverse | CTCCTGGAAGATGGTGATGGGAT |
| SLC7A1 | forward | ATGGGTGGAAACGCTGATGATAC |
|  | reverse | ACCTTGCCTGTTAAGTCTGGGTG |
| SLC7A2-2A | forward | TTTAACACTTATGATGCCGTACTACCT |
|  | reverse | GCAACTGGTGACTGCCTCTTACT |
| SLC7A2-2B | forward | ATGCCTCGTGTAATCTATGCTATG |
|  | reverse | ACTGCACCCGATGATAAAGTAGC |
| SLC7A3 | forward | CACATCTCTAAGACTCTGCAGGGG |
|  | reverse | CTCTCCTATGTCATTGGTACAGCCAG |
| SLC7A4 | forward | GCTGCGTGCTTGTCTTTGGGAACT |
|  | reverse | TTCAGCATGAGGCAGATGTTGAGG |
| SLC7A6 | forward | TTCTTGACAGGCAGTGGCGTGAT |
|  | reverse | CTGGGACTGGCTGGTGTTAGGGA |
| SLC7A7 | forward | GAAGGAGGAGCATCAGACCA |
|  | reverse | CCCAGTTCCGCATAACAAAG |
| SLC7A8 | forward | GGCATCTCTCTTCCTAATG |
|  | reverse | GCCAATGCTCTCCTCAGT |
| SLC7A9 | forward | ttgacctctgtaccttctgga |
|  | reverse | tcaagcagtccttccacctc |
| SLC7A10 | forward | TGGCTGGAACTTCCTCAACT |
|  | reverse | GATGGCACGAGGTAGGTTCT |
| SLC7A11 | forward | ACCATCAGTGCGGAGGAG |
|  | reverse | AAGATCGGGACTGCTAATGAGA |
| SLC7A13 | forward | GGAAAAGTGATAAAGCTATAGATCCAA |
|  | reverse | GGCTGGCATGATCTGATTCAG |
| SLC7A14 | forward | TCCCTCAAGATTGTCAGCAA |
|  | reverse | CACCACCTTCTTGATGTCATC |
